# Accuracy of p57^KIP2^ compared with genotyping for the diagnosis of complete hydatidiform mole: protocol for a systematic review and meta-analysis

**DOI:** 10.1186/s13643-016-0349-7

**Published:** 2016-10-04

**Authors:** Jose Mauro Madi, Antonio Rodrigues Braga, Machline Paim Paganella, Isnard Elman Litvin, Eliana Márcia Da Ros Wendland

**Affiliations:** 1 School of Medicine, Center for Biological and Health Sciences – CCBS, Caxias do Sul University - UCS, Rua Francisco Getúlio Vargas, 1130, Bloco S, Caxias do Sul, RS CEP 95070-560 Brazil; 2 Maternity School, Federal University of Rio de Janeiro – UFRJ, Rua das Laranjeiras, 180, Rio de Janeiro, RJ CEP 22240-003 Brazil; 3 School of Medicine, Federal University Fluminense – UFF, Niterói, RJ Brazil; 4 HIV/AIDS Research Laboratory – LPHA, Center for Biological and Health Sciences – CCBS, Caxias do Sul University – UCS, Rua Francisco Getúlio Vargas, 1130, Bloco S, Sala 315, Caxias do Sul, RS CEP 95070-560 Brazil; 5 Department of Public Health, Federal University of Health Science of Porto Alegre - UFCSPA, Rua Sarmento Leite, 245, Porto Alegre, RS CEP 90050-170 Brazil

**Keywords:** Complete hydatidiform mole, p57 immunohistochemistry, Molecular genotyping, Protocol, Systematic review, Meta-analysis

## Abstract

**Background:**

Distinguishing hydatidiform moles (HMs) from non-molar specimens and the subclassification of HM are important because complete hydatidiform mole (CHM) is associated with an increased risk of gestational trophoblastic neoplasia. However, diagnosis based solely on morphology has poor interobserver reproducibility. Recent studies have demonstrated that the use of p57^KIP2^ immunostaining improves diagnostic accuracy for CHM.

**Methods:**

We will conduct a systematic review of prospective and retrospective studies to evaluate the accuracy of p57^KIP2^ immunostaining compared with molecular genotyping for the diagnosis of CHM. A high-sensitivity search strategy will be employed in MEDLINE, EMBASE, LILACS, The Grey Literature Report, OpenGrey, OAIster, and Cochrane CENTRAL. Two reviewers will independently screen all identified references for eligibility and extract data. The methodological quality and bias of the included studies will be assessed according to the Quality Assessment of Diagnostic Accuracy Studies (QUADAS-2) tool, and the overall quality of evidence will be assessed using the Grading of Recommendations Assessment, Development, and Evaluation (GRADE) approach. If a meta-analysis is possible, pooled estimates of sensitivity, specificity, and positive and negative likelihood ratios will be calculated using bivariate random-effects models. Statistical heterogeneity will be evaluated with *I* ^2^ statistics and explored through sensitivity analysis.

**Discussion:**

There is considerable overlap between the histological features of molar and non-molar pregnancies and between complete and partial HMs, which results in significant interobserver variability in the diagnosis of CHM and its mimics. Therefore, molecular techniques are used to correctly diagnosis and treat CHM. However, these molecular diagnostic methods are technically difficult to perform, relatively costly, and unavailable in most pathology laboratories. According to our results, p57^KIP2^ immunostaining appears to be a practical and accurate adjunct for the diagnosis of CHM and its mimics because this technique is relatively simple, reliable, cost-efficient, and rapid. This systematic review will help to determine whether p57^KIP2^ immunostaining is an adequate alternative diagnostic test for CHM.

**Systematic review registration:**

PROSPERO CRD42015024181

**Electronic supplementary material:**

The online version of this article (doi:10.1186/s13643-016-0349-7) contains supplementary material, which is available to authorized users.

## Background

### Description of the condition

Hydatidiform mole (HM) is an abnormal gestational condition characterized by significant hydropic enlargement and variable trophoblastic proliferation involving part or all of the chorionic villi. Histopathological examination remains the basis for the diagnosis of HM; however, the diagnosis and classification of HM has become increasingly difficult because HMs are now commonly evacuated at an earlier stage and do not satisfy the well-established classic morphological features [1]. Previous studies have demonstrated that the diagnosis of HM based on morphology alone is subject to interobserver variability and therefore suboptimal diagnostic reproducibility [2, 3]. Differentiating a molar pregnancy from non-molar specimens and the classification of HM as complete hydatidiform mole (CHM), partial hydatidiform mole (PHM), early CHM (eCHM) or hydropic abortion (HA) is important for both clinical practice and investigational studies because the risk of persistent gestational trophoblastic disease, including choriocarcinoma, is significantly higher after a pregnancy affected by CHM (10–30 %) or PHM (0.5–5 %) compared with any other pregnancy [2–4].

The p57^KIP2^ gene is paternally imprinted and maternally expressed, and the presence of its protein product serves as a surrogate marker for the nuclear maternal genome. CHM is the only type of conceptus lacking a maternal contribution, and p57^KIP2^ immunostaining is accordingly absent, whereas it is present in CHM mimics [4].

### Description of the index test

The index test will consist of p57^KIP2^ immunostaining. P57^KIP2^ immunostaining is an in situ technique performed on paraffin-embedded tissue. The results usually are easy to interpret. P57 is a cyclin-dependent kinase inhibitor and tumour suppressor gene located on chromosome 11p15.5. The lack of p57^KIP2^ activity can lead to a loss of cell cycle control, which results in the abnormal proliferation and differentiation of trophoblasts in CHM. As expected, a lack of the p57^KIP2^ protein product has been demonstrated in immunohistochemistry (IHC)-based studies on CHMs, but not in those on non-CHMs, which suggests that p57^KIP2^ IHC can be helpful for distinguishing CHM from its mimics. The p57^KIP2^ gene (CDKN1C) is a strongly paternally imprinted gene that is expressed only by the maternal allele in most tissues and is involved in implantation. P57^KIP2^ immunostaining is absent in CHM due to the lack of a maternal genome [1].

Explanations for the negative expression of p57^KIP2^ in discordant cases include misdiagnosed CHM, false-negative results in a non-CHM patient, or a lack of staining due to loss of antigenicity. False-positive staining is attributed to the retention of maternal chromosome 11, which is a phenomenon that is rarely seen in CHM [1]. Immunostaining is assessed in the nuclei of the villous mesenchymal cells (VMC) and cytotrophoblasts. Positive immunostaining is considered to exist when more than 10 % of the nuclei of the cytotrophoblasts and VMC are stained. Immunostaining of extravillous trophoblast cells, trophoblastic cells within cell columns or islands, and interstitial trophoblasts at the maternal-foetal junctional zones are observed as internal positive controls. Syncytiotrophoblastic cells are used as negative controls [1, 2, 4]. It was recently demonstrated that p57^KIP2^ immunostaining can be helpful for refining the diagnosis of some morphologically challenging cases and for the detection of androgenetic cell lines in mosaic/chimeric conceptions [1]. There are rare examples where the morphology and immunophenotype (p57-negative) of CHM occur in patients with familial recurrent HMs associated with mutations in NLRP7 (NALP7) or KHDC3L (C6orf221) [4].

### Description of the standard test

Genotyping, which is accomplished through PCR amplification of short tandem repeat loci, is particularly valuable for the diagnosis of HM because it allows the specific distinction of CHM, PHM, eCHM, and HA. Through genotyping, CHM is diagnosed based on the finding of purely androgenetic alleles. The vast majority of CHMs are characterized by androgenetic diploidy (two sets of paternal chromosome complements without a maternal chromosome complement); however, a small subset can exhibit androgenetic tetraploidy (genotyping does not specifically distinguish examples of diploidy from tetraploidy because peak heights do not indicate the actual DNA content) [4]. Analysis of nuclear DNA microsatellite polymorphisms is particularly well suited for the diagnosis of MHC because it is capable of determining the number and parental origin of alleles. Molecular diagnostic methods such as genotyping are limited because they are technically difficult, relatively costly, and not universally available. In addition, ploidy analysis does not differentiate between CHM and HA because both are diploid. It has been reported that CHM results from the fertilization of an enucleated egg by a haploid sperm, followed by duplication of the sperm genome (homozygous). However, in approximately 10–25 % of cases, the enucleated egg is fertilized by two sperm cells (heterozygous). In some cases diagnosed using microsatellite genotyping, androgenic CHM can be misdiagnosed as biparental mole, containing maternal alleles, due to possible contamination of the molar tissue by maternal tissue that was inadvertently sent for analysis. Analyses of genotyping results can be difficult to interpret if abundant tissue of maternal origin is present. Genotyping analysis plays an important role in the diagnosis of challenging cases with unusual p57^KIP2^ results [1, 4]. The goals of this systematic review are to generate new quantitative evidence for clinicians and to establish the accuracy of p57^KIP2^ IHC compared with genotyping for the identification of CHM.

## Methods

### Design

The methodological approach for evidence searching and synthesis described in this protocol will conform to the Cochrane Collaboration’s methods for assessing diagnostic test accuracy [5]. We will also follow the recommendations of the Preferred Reporting Items for Systematic Review and Meta-Analysis Protocols (PRISMA-P) included as an additional file (Additional file 1) [6].

The study is registered at PROSPERO, the International Prospective Register of Systematic Reviews, at the University of York (CRD42015024181).

### PIRO question

For analyses of the diagnostic accuracy of research questions, the acronym PIRO is used, corresponding to P (population), I (index test), R (reference standard), and O (outcome).

The definitions of the components of the PIRO acronym for this systematic review are as follows:

- Population: women of reproductive age (14–50 years)
- Index test: p57^KIP2^ immunohistochemistry
- Reference standard: genotyping
- Outcome: MHC idenification.

### Search methods for identifying studies

Keywords and Medical Subject Headings related to HM, p57^KIP2^ and molecular genotyping will be used alone or in combination (together with synonyms and closely related words) to retrieve the relevant articles.

We will conduct searches in the Excerpta Medica Database (EMBASE), Centro Latinoamericano y del Caribe de Información en Ciencias de la Salud (LILACS), Medical Literature Analysis and Retrieval System Online (MEDLINE), Cochrane Central Register of Controlled Trials (CENTRAL), and Web of Science. We will also screen the reference lists of relevant studies and reviews for additional articles and will search the grey literature websites The Grey Literature Report, OpenGrey, and the Open Archives Initiative (OAIster). If necessary (in the case of unclear data, missing data or extractable data), we will attempt to contact the corresponding authors of the included studies for missing data and for clarification. The search strategy developed for MEDLINE (see Additional file 2) will be adapted for the other databases. There will be no language or publication year restriction.

### Eligibility criteria for the included studies

Any cross-sectional study, case series, case-control study, cohort study, or clinical trial that evaluates the accuracy of p57^KIP2^ immunostaining for the diagnosis of CHM compared with genotyping will be included. Case reports, narrative reviews, and expert opinions will be excluded. Animal testing will be excluded.

### Data collection

Two independent researchers will evaluate the titles and abstracts arising from the combined search and will independently extract all data from the retrieved articles using a predefined data extraction sheet. A third author will adjudicate any discrepancies.

In the case of duplicate publications or more than one publication from a preliminary study, we will attempt to maximize the use of the information by simultaneously evaluating all of the available data, but we will not include the same group of patients in the analysis more than once.

The data will be extracted in the form of a data sheet specifically developed for this analysis (Tables 1 and 2). The following information will be extracted from each study, with the possibility of adding further information during the extraction process when appropriate:

- Study characteristics: title, author, country, design, language of publication, year of publication, sample size, and number of centres
- Population characteristics: total number of patients, number of patients in groups for comparison, and age of the patients
- Index test: type of test and diagnostic criteria
- Standard test: type of test and diagnostic criteria
- Outcomes: number of true positives, false positives, true negatives, and false negatives, sensitivity and specificity, negative predictive value (NPV) and positive predictive value (PPV), and the positive likelihood ratio (LR+) and negative likelihood ratio (LR−)

**Table 1:** Characteristics of the studies included

#	Author	Year	Country	Language	Study design	Age	CHM/IHC	CHM/Geno	# of centers
1									
2									
3									
4									

*CHM* complete hydatidiform mole, *IHC* immunohistochemistry, *Geno* genotyping

**Table 2:** Outcomes

#	Author	N	TP	FP	TN	FN	S	E	NPV	PPV	LR+	LR−
1												
2												
3												
4												

*TP* true positive, *FP* false positive, *TN* true negative, *FN* false negative, *S* sensitivity, *E* especificity, *NPV* negative predictive value, *PPV* positive predictive value, *LR* likelihood ratio

If there are any missing or insufficient data in the included studies, we will contact the corresponding authors of the studies via email to obtain additional information. When more than one threshold is available, all data will be recorded. A sensitivity analysis will be conducted to assess the impact of including studies with 20 % or more missing data.

### Risk of bias assessment

We will assess the quality of the studies using the Quality Assessment of Diagnostic Accuracy Studies (QUADAS-2) tool [7]. If necessary, QUADAS-2 will be adapted to fit different study designs included in accordance with our research question. Grading of Recommendations Assessment, Development, and Evaluation (GRADE) will be used to rate the quality of the body of evidence retrieved in the search [8].

### Outcomes

The primary outcome will be the diagnostic accuracy of p57^KIP2^ immunostaining for the diagnosis of CHM, which will be described based on sensitivity and specificity, negative and positive predictive values, and positive and negative likelihood ratios wherever possible.

### Statistical analysis

Where the data permit, we will compare the index test against the reference test. For each study, we will extract the number of true positives, true negatives, false positives, and false negatives. When the raw data are provided, contingency tables will be built to display the results of the tests. The test results will be treated as positive or negative for the cut-off values of the index test. The sensitivity, specificity, positive predictive value, false-positive rate, and positive likelihood ratio will be calculated from the cut-off values of the index test. Forest plots will be generated to illustrate sensitivity and specificity and the 95 % confidence intervals (CI). The diagnostic odds ratio (DOR) and the area under the curve (AUC) of the summary ROC (summary receiver operating characteristic (SROC)) will be calculated because the summary statistics indicate the power of the overall assessment for each of the two tests. A hierarchical model, using the correlation between sensitivity and specificity across the studies to provide a summary of the model, will be employed to calculate the pooled estimation using the R software 3.3.1 packages metafor [9], mada [10], and HSROC [11]. The choice between the bivariate random-effects model [12] and the HSROC model from Rutter e Gatsonis [13] will be based on the presence of different thresholds. Both approaches can be used to compute estimates of the summary ROC curve and the average operating point and allow us to determine the extent of heterogeneity in the estimated pooled measure. If heterogeneity is detected, we will conduct a subgroup analysis and meta-regression to evaluate the impact of the covariates in the pooled estimation. The two models are mathematically equivalent when no covariates are included in the model.

The magnitude of heterogeneity will be assessed using Cochran’s Q statistic and Higgins *I* ^2^ statistic, where an *I* ^2^ greater than 50 % indicates the presence of significant heterogeneity. The *I* ^2^ statistic will be calculated according to the following equation: *I* ^2^ = 100 % × (*Q* − *df)*/Q, where *Q* is the Cochran heterogeneity statistic [14]. If quantitative synthesis is not appropriate, a descriptive analysis might be undertaken.

We will perform a sensitivity analysis to examine the effect of sample size and missing data on the results of the review. If there are adequate studies (no less than three studies), we will conduct a sensitivity analysis to check the robustness of the conclusions and assess the impact of the methodological quality.

The presence of publication bias will be assessed by performing a regression of lnDOR and the effective sample size (ESS) based on methods described by Deeks et al. [15].

## Discussion

There is considerable overlap in histological features between molar and non-molar pregnancies and between CHMs and PHMs, which results in significant interobserver variability in the diagnosis of HM and its mimics. Therefore, correct diagnosis of these difficult cases may require molecular techniques that examine the differences in DNA content between CHM and PHM, including flow or image cytometric DNA analysis, chromosome in situ hybridization, polymerase chain reaction-based genotyping, or HLA typing. However, these molecular diagnostic methods are technically difficult to perform, relatively costly, and unavailable in most pathology laboratories [1].

Banet et al. established that immunohistochemical analysis of p57^KIP2^ expression is highly correlated with genotyping results and demonstrated that CHM is almost always p57-negative, with only rare examples (0.5 %) displaying aberrant (positive) p57^KIP2^ expression, which is attributable to retention of the maternal copy of chromosome 11. CHMs are androgenetic conceptions by definition, and the vast majority are monospermic (85 %) [4].

The rare examples of aberrant p57^KIP2^ expression in both CHM and PHM can be correctly classified using genotyping. The findings of Banet et al. demonstrated that p57^KIP2^ IHC is extremely reliable for the diagnosis of CHM. Therefore, the algorithmic approach for the diagnosis of HM proposed in this study advocates that p57^KIP2^ results be used to triage cases for genotyping because this technique provides a highly reliable method for accurately diagnosing CHM in routine practice using a single immunohistochemical stain, with very little risk of misclassification of CHM. Consequently, genotyping for CHM is not necessary in routine practice and can be reserved for problematic cases, such as when p57^KIP2^ immunostaining is suboptimal or unsatisfactory or when there is a discrepancy between morphology and p57^KIP2^ results. One exception would be the case of recurrent HM, which raises the possibility of familial biparental HM. Patients with this disorder can have multiple/recurrent CHMs that are morphologically, immunophenotypically, and clinically similar to conventional CHMs; specifically, they are p57-negative and appear to show a similar risk of persistent gestational trophoblastic disease but are characterized by biparental diploidy rather than androgenetic diploidy [4].

Therefore, genotyping is useful in any patient with recurrent HMs to determine if they represent the familial form. It is important to recognize that the biparental form exists so that the genotyping results of biparental diploidy are not used to reject a diagnosis of CHM when the morphology and/or p57^KIP2^ results support a diagnosis of CHM [4].

Some studies [1, 4] have confirmed that p57^KIP2^ immunostaining is a practical and accurate adjunct for the diagnosis of CHM and its mimics because this technique is a relatively simple, reliable, cost-efficient, and rapid procedure. Therefore, the most ideal method for correctly classifying all HMs and non-molar specimens is a combined approach that includes the correlation of morphological features, p57^KIP2^ IHC, and molecular genotyping [4]. This combined approach is particularly important when evaluating difficult and challenging cases with discordant positive p57^KIP2^ staining, when molecular techniques are still necessary [1]. The findings of Banet et al. also confirmed that p57^KIP2^ IHC analysis is useful for identifying androgenetic/biparental mosaic/chimeric conceptions, which include uniformly androgenetic/biparental mosaic specimens without molar features (probably early forms of placental mesenchymal dysplasia, which is characterized by androgenetic/biparental mosaicism and a lack of trophoblastic hyperplasia), androgenetic/biparental mosaic specimens with a molar component (typically CHMs), and twin gestations composed of CHM and non-molar specimen components. Recognition of the discordant and divergent staining patterns in these specimens is key to correctly interpreting these complex specimens and is necessary for specific microdissection of the different components to assure accurate molecular genotyping [4].

Therefore, we decided to perform this systematic review and meta-analysis using the most definitive method to assess the accuracy of p57^KIP2^ IHC compared with molecular genotyping for the diagnosis of CHM.

## Additional files

Additional file 1: PRISMA-P recommendations. (DOC 83 kb)
Additional file 2: Search strategy for MEDLINE/PUBMED. (DOCX 11 kb)

## Abbreviations

AUC: Area under the curve
CENTRAL: Cochrane Central Register of Controlled Trials
CHM: Complete hydatidiform mole
CI: Confidence interval
DNA: Deoxyribonucleic acid
DOR: Diagnostic odds ratio
eCHM: Early CHM
EMBASE: Excerpta Medica Database
GRADE: Grading of Recommendations Assessment, Development and Evaluation
HA: Hydropic abortion
HLA: Human leukocyte antigen
HM: Hydatidiform mole
IHC: Immunohistochemistry
LILACS: *Centro Latinoamericano y del Caribe de Información en Ciencias de la Salud*
LR: Likelihood ratio
MEDLINE: Medical Literature Analysis and Retrieval System Online
NPV: Negative predictive value
OAIster: Open Archives Initiative
PHM: Partial hydatidiform mole
PIRO: Population, index test, reference standard test, outcome
PPV: Positive predictive value
PRISMA-P: Preferred Reporting Items for Systematic Review and Meta-Analysis Protocols
QUADAS-2: Quality Assessment of Diagnostic Accuracy Studies
ROC: Receiver operating curve
SROC: Summary ROC
VMC: Villous mesenchymal cells
