# Supplementary material for: Accuracy of p57KIP2 compared with genotyping for the diagnosis of complete hydatidiform mole: protocol for a systematic review and meta-analysis
Source: Syst Rev. 2016 Oct 4;5:169. doi: 10.1186/s13643-016-0349-7 (PMC5050667; doi:10.1186/s13643-016-0349-7)
Supplement: Additional file 2: — Search strategy for MEDLINE/PUBMED. (DOCX 11 kb) [file 13643_2016_349_MOESM2_ESM.docx]

**ADDITIONAL FILE 2**

**MEDLINE/PUBMED Search Strategy**

(("immunohistochemistry"[MeSH Terms] OR (((("Cyclin-Dependent Kinase Inhibitor p57"[Mesh] OR p57[All Fields]) OR p57kip2[All Fields]) OR p57-protein[All Fields]) OR (p57[All Fields] OR p57'[All Fields] OR p57's[All Fields] OR p570[All Fields] OR p57053[All Fields] OR p570s[All Fields] OR p571[All Fields] OR p571168201r67073[All Fields] OR p5713382678v12pp[All Fields] OR p5713k1573m2731l[All Fields] OR p5714u1815446161[All Fields] OR p571a[All Fields] OR p571s[All Fields] OR p572[All Fields] OR p5722[All Fields] OR p572287h34338x4p[All Fields] OR p572377204574584[All Fields] OR p572423l532487q5[All Fields] OR p5724r52m1vpr124[All Fields] OR p572j2417178l5rj[All Fields] OR p572s[All Fields] OR p572y[All Fields] OR p573[All Fields] OR p57319t454366005[All Fields] OR p5731x67416333k1[All Fields] OR p5732112x4n1t558[All Fields] OR p573632586212u32[All Fields] OR p573nv7r3654252g[All Fields] OR p573t[All Fields] OR p573v271v881348v[All Fields] OR p574[All Fields] OR p574h[All Fields] OR p574l[All Fields] OR p574r[All Fields] OR p574s[All Fields] OR p574w11h3l218u57[All Fields] OR p575[All Fields] OR p575031m64037385[All Fields] OR p575387510xh361w[All Fields] OR p57578182150411m[All Fields] OR p575l[All Fields] OR p575s[All Fields] OR p576[All Fields] OR p576628210585728[All Fields] OR p57678[All Fields] OR p5767806661p1810[All Fields] OR p57686120n8uq872[All Fields] OR p577[All Fields] OR p577086v820161w2[All Fields] OR p5771[All Fields] OR p57716[All Fields] OR p57740[All Fields] OR p57745[All Fields] OR p577532694u41828[All Fields] OR p57780r27w0381m4[All Fields] OR p57785[All Fields] OR p577852645w44865[All Fields] OR p5779[All Fields] OR p577a[All Fields] OR p577del[All Fields] OR p577delinsh[All Fields] OR p577l[All Fields] OR p577s[All Fields] OR p578[All Fields] OR p578378873136641[All Fields] OR p578391613u73q17[All Fields] OR p5784j3vr36r6801[All Fields] OR p5786831326254q8[All Fields] OR p578s[All Fields] OR p579[All Fields] OR p579p[All Fields] OR p57a[All Fields] OR p57akt[All Fields] OR p57as3[All Fields] OR p57b[All Fields] OR p57c[All Fields] OR p57f8bcq[All Fields] OR p57fyn[All Fields] OR p57g4[All Fields] OR p57k[All Fields] OR p57k1p2[All Fields] OR p57kip[All Fields] OR p57kip2[All Fields] OR p57kip22[All Fields] OR p57kip2a[All Fields] OR p57kip2and[All Fields] OR p57lz[All Fields] OR p57m0142t25651j8[All Fields] OR p57myc[All Fields] OR p57n2[All Fields] OR p57ntr[All Fields] OR p57p[All Fields] OR p57pgp1[All Fields] OR p57rettpc[All Fields] OR p57s[All Fields] OR p57t85623m90t72g[All Fields] OR p57twl22ix[All Fields] OR p57v[All Fields]))) AND ((((((("Genotyping Techniques"[Mesh] OR genotyping[All Fields]) OR (("dna"[MeSH Terms] OR "dna"[All Fields]) AND genotyping[All Fields])) OR (dna ploid[All Fields] OR dna ploidia[All Fields] OR dna ploidianalyse[All Fields] OR dna ploidie[All Fields] OR dna ploidiegrad[All Fields] OR dna ploidies[All Fields] OR dna ploidii[All Fields] OR dna ploiditet[All Fields] OR dna ploidity[All Fields] OR dna ploidy[All Fields])) OR "DNA ploidy"[All Fields]) OR "microsatellite DNA genotyping"[All Fields]) OR "microsatellite genotyping"[All Fields]) OR "microsatellite DNA"[All Fields])) AND (((((("Hydatidiform Mole"[Mesh] OR ("hydatidiform mole"[MeSH Terms] OR ("hydatidiform"[All Fields] AND "mole"[All Fields]) OR "hydatidiform mole"[All Fields])) OR "gestational trophoblastic disease"[All Fields]) OR "partial hydatidiform mole"[All Fields]) OR "trophoblastic disease"[All Fields]) OR "complete hydatidiform mole"[All Fields]) OR ("gestational trophoblastic disease"[MeSH Terms] OR ("gestational"[All Fields] AND "trophoblastic"[All Fields] AND "disease"[All Fields]) OR "gestational trophoblastic disease"[All Fields]))
